# Quality of life after aortic valve repair is similar to Ross patients and superior to mechanical valve replacement: a cross-sectional study

**DOI:** 10.1186/s12872-016-0236-0

**Published:** 2016-04-02

**Authors:** Pavel Zacek, T. Holubec, M. Vobornik, J. Dominik, J. Takkenberg, J. Harrer, J. Vojacek

**Affiliations:** Department of Cardiac Surgery, Charles University in Prague, Faculty of Medicine and Faculty Hospital, Sokolska 581, 50005 Hradec Kralove, Czech Republic; Department of Cardiac Surgery, Kerckhoff Heart and Lung Center, Bad Nauheim, Germany; Department of Cardio-Thoracic Surgery, Erasmus MC, Rotterdam, The Netherlands

**Keywords:** Quality of life, Aortic valve, Valve-sparing, Valve replacement, Anticoagulation, Ross procedure

## Abstract

**Background:**

In patients after aortic valve surgery, the quality of life is hypothesized to be influenced by the type of the valve procedure. A cross-sectional study on the postoperative quality of life was carried out in patients after aortic valve-sparing surgery (with regards to the age of the patient), Ross procedure and mechanical aortic valve replacement.

**Methods:**

Quality of life was studied in 139 patients after aortic valve surgery divided into four study groups (Y – aortic valve-sparing procedure at the age below 50 years, mean age 36.2 years; O – aortic valve-sparing procedure at the age 50 years and over, mean age 59.2 years; R – Ross procedure, mean age 37.8 years and M – mechanical aortic valve replacement at the age below 50 years, mean age 39.2 years). SF-36 Short Form and valve-specific questionnaires were mailed to the patients after 6 months or later following surgery (median 26.9 months).

**Results:**

In SF-36, the younger aortic valve repair patients and the Ross patients scored significantly better in 4 of 4 physical subscales and in 2 of 4 mental subscales than the older aortic valve repair and mechanical valve replacement patients. In the valve-specific questionnaire; however, all 3 groups free of anticoagulation (Y, O, and R) displayed greater freedom from negative valve-related concerns.

**Conclusions:**

Postoperative quality of life is influenced by the type of aortic valve procedure and is negatively linked with mechanical prosthesis implantation and long-term anticoagulation. Aortic valve-sparing strategy should be considered in cases with suitable valve morphology due to favorable clinical results and beneficial impact on the long-term quality of life.

## Background

Aortic valve-sparing procedures (AVS) represent currently an appealing surgical strategy in the treatment of aortic regurgitation (AR) with/without dystrophic aortic root dilation. Although bearing an inherent uncertainty of the durability of the repair the main rationale for attempting valve-sparing surgery in patients with suitable morphology is avoidance of the classical risks related to the valve replacement devices. This applies especially for younger patients who otherwise would mostly be candidates for mechanical valve with a lifelong burden of coumadin administration. As a result of accumulated experience, a certain degree of standardization and simplification of surgical valve-sparing techniques allows a safe and predictable result. Low operative risk makes therefore the AVS eligible also for older population of patients who otherwise are believed to benefit from a bioprosthetic valve replacement.

Several studies report favorable early and midterm clinical outcomes after AVS surgery [1–10]. Quality of life (QoL), on contrary, is a complex and diverse value which may poorly correlate with the clinical data. It is hypothesized that, besides an improvement in clinical outcome, there is a positive impact on QoL desired in patients after AVS, as they are free of concerns related to coumadin medication. We conducted a single center cross-sectional survey to assess and compare the quality of life in adult patients undergoing AVS, Ross procedure and mechanical valve replacement.

## Methods

The study was approved by the Institutional Review Board of the University Hospital, Hradec Kralove. Between 2006 and 2012, a total of 1424 aortic valve procedures were performed at our institution, comprising aortic valve replacement with/without replacement of the ascending aorta, aortic valve-sparing procedures and Ross procedures. The age of the patients ranged between 19 and 86 years. The time frame for patients’ recruitment was deduced from the period of institutional started-up aortic valve-sparing program (since 2007) and Ross program (since 2009). To gather a comparable number of mechanical aortic valve replacement (AVR) patients, these patients were enrolled already since 2006.

To be able to identify the possible impact of the chosen type of aortic valve procedure on QoL in comparable groups, all consecutive patients after valve-sparing and Ross procedures were enrolled. With regard to the younger age profile of the Ross patients the patients after AVS were divided into two age groups (delineated by the age of 50). For mechanical AVR, all consecutive patients below the age of 50 and having an isolated aortic procedure (valve w/or w/o root surgery) were enrolled. Thus, the quality of life was studied in a total of 139 patients after aortic valve surgery divided into four study groups:

1. Y – 36 patients after aortic valve-sparing procedure, including aortic root procedure. Inclusion criteria: age below 50 years (mean age 36.2 years, operated 2/2008–5/2012). From the original number of 41, 2 patients did not respond the questionnaires and 3 patients were excluded from the survey: one patient died during follow-up from non-cardiac reason and two were reoperated for the valve repair failure.
2. O - 52 patients after aortic valve-sparing procedure, including aortic root procedure. Inclusion criteria: age 50 years and over (mean age 59.2 years, operated 11/2007–6/2012). From the original number of 56, 4 patients were excluded: one patient died 1.5 month due to stroke and 3 were reoperated for the valve repair failure.
3. R – 22 patients after Ross procedure (mean age 37.8 years, operated between 11/2009 and 8/2012). No exclusion criteria.
4. M – 29 patients after AVR with a mechanical prosthesis. Inclusion criteria: elective aortic valve replacement - isolated or combined with ascending aortic procedure at the age below 50. Exclusion criteria: left ventricular ejection fraction below 50 %, coronary artery disease or other cardiac procedure. Mean age 39.2 years. Operated 3/2006–9/2012. From the original number of 31, 2 patients did not respond the questionnaires.

The type of the aortic valve surgery in each patient was dictated by the valve and root pathomorphology with a clear aim to prioritize the valve salvage. A conservative aortic valve surgery was therefore performed in all patients with pure AR in whom the pre-operative echocardiography analysis and intraoperative valve assessment allowed for the repair. The patients with pure AR not eligible for repair (restrictive leaflet morphology) or concomitant aortic valve stenosis (AS) were offered valve replacement with a mechanical valve or a Ross procedure. Ross procedure was suggested to patients who clearly disapproved the coumadin solution, typically younger individuals with an active life-style. All patients were informed in detail about all the surgical modalities and their relevant advantages and drawbacks. The patients themselves made the final decision for a given procedure. Aortic valve- sparing procedures were performed by 3 surgeons whereas the Ross procedure was performed by a single surgeon.

Quality of life was assessed by means of mailed questionnaires to be filled without additional counseling. An informed consent was obtained as an integral part of the questionnaire. The questionnaires were sent to the patients no sooner than 6 months after the surgery. Short Form Health Survey (SF-36) [11] and valve-specific questionnaire, as published by Perchinsky [12], were used as survey tools. In general, in SF-36 there were 36 items analyzed in 8 subscales (physical functioning, physical role, bodily pain, general health, vitality, social functioning, emotional role, and mental health). Valve-specific questionnaire comprised of 8 questions on specific features of life after valve surgery (question on being disturbed by the sound of valve prosthesis was omitted as confusing in Ross and AVS patients).

As mentioned above, 5 patients after AVS who required reoperation for the valve repair failure were not sent the questionnaires due to potential bias. The reasons of failure were miscellaneous: progressive restriction of the leaflets, tearing of the cusp plication, absence of annular stabilization in isolated cusp repair, giant-cell aortitis). Reoperations were performed after a mean interval of 21 months (7–60); all valves were replaced with a zero mortality.

### Statistical analysis

Continuous variables were described as mean ± SD and categorical variables as counts and absolute and relative frequencies. The comparisons between groups were performed by Fisher´s Exact Test for categorical variables and for SF-36 scoring results for intervals 100–90, 89–70, 69–50, 49–0. A *p*-value of 0.05 was used for determining significant difference. NCSS statistical software was used (NCSS, LLC Kaysville, Utah, USA).

## Results

### Patient population results

Patients’ demographic, preoperative and postoperative clinical data are summarized in Table 1.

**Table 1:** Patients’ demographic and clinical data (preoperative and follow-up)

	Y (repair)	O (repair)	Ross	M (mechAVR)
Preoperative:	*n* = 36	*n* = 52	*n* = 22	*n* = 29
Age (years), ø ± SD	36.3 ± 6.1	59.2 ± 7.7	37.8 ± 11.9	39.7 ± 7.3
Males (%)	78	70	83	69
NYHA class (%)				
I	50	23	48	41
II	39	55	39	45
III	11	23	13	14
IV	3	0	0	0
Bicuspid Ao-valve (%)	78	46	35	79
Unicuspid Ao-valve (%)	8	0	61	7
Marfan sy (%)	8	0	0	0
Art. hypertension (%)	39	64	22	31
Diabetes (%)	6	18	0	0
EF (%)	61 ± 8	58 ± 11	65 ± 8	65 ± 6
AR preop. (%)				
Grade 0	3	2	0	7
1	11	9	13	14
2	6	9	4	14
3	8	15	4	3
4	72	64	78	62
AS severe (%)	0	0	36	45
At latest follow-up:				
NYHA class (%)				
I	94	75	100	100
II	6	25	0	0
III	0	0	0	0
IV	0	0	0	0
EF (%)	62 ± 6	61 ± 9	63 ± 5	65 ± 7
AR postop. (%)				
Grade 0	47	38	22	0
1	32	38	61	0
2	12	19	9	0
3	0	2	4	0
4	3	4	0	0

*AR* aortic regurgitation, *AS* aortic stenosis, *NYHA* New York Heart Association functional classification, *EF* left ventricular ejection fraction

Patient age (excl. group O), sex, NYHA functional class, left ventricular ejection fraction (EF) and advanced degree of aortic regurgitation (AR) did not differ between the groups. A high proportion of bicuspid aortic valve morphology was marked in groups Y and M, while unicuspid valvular morphology was extraordinary frequent in the group R. At the follow-up, all groups achieved excellent functional improvement in terms of NYHA class, residual AR and EF, irrespective of the surgical procedure.

### Procedural details

All procedures were performed via standard sternotomy approach. Surgical strategy in valve-sparing groups (group Y and O) was tailored according to the individual patient’s morphology to correct the diameters of ventriculo-aortal and sino-tubular junctions and to obtain equal effective height of the aortic cusps (9–10 mm).

Surgical techniques involved cusp plication (25 and 28; Y and O resp.), suture of the cleft in a bicuspid aortic valve (8 and 6), or isolated supracoronary aortic replacement (8 and 17). Complex root repair was required in 38 patients, accomplished by root reimplantation technique (David procedure) (9 pts.; 2 and 7; Y and O resp.), root remodeling technique (Yacoub procedure) (27 pts.; 11 and 16) combined with Extra Aortic annuloplasty ring (Coronéo Inc., Montreal, QC, Canada) (23 pts.; 11 and 12). Additional cardiac procedures were performed in 24 pts. – Table 2.

**Table 2:** Surgical techniques used for aortic valve repair and additional procedures

	Y (repair)	O (repair)
Repair techniques:	*n* = 36	*n* = 52
Central plication of the leaflet	25	28
Suture of the cleft	8	6
Supracoronary aortic replacement	8	17
Yacoub procedure	11	16
David procedure	2	7
Annuloplasty ring	11	12
Additional procedures:		
CABG	0	4
Mitral valve repair	3	4
Hemiarch replacement	1	5
MAZE	0	6
Tricuspid valve repair	0	1

*CABG* coronary artery bypass grafting, *MAZE* cryosurgical ablation for atrial fibrillation

### Postoperative quality of life

SF-36 Short Form QoL questionnaire was completed by 139 patients (Y = 36, O = 52, R = 22, M = 29) at a median of 26.9 months postoperative (range 6–73 months). The questionnaire could not be obtained from 4 patients (two after AVS and two after mechanical AVR). In general, across all 8 subscales there were marked trends for better performance of the group Y (younger aortic valve repair) and R (Ross) against worse performance of the group O (older aortic valve repair) and M (mechanical AVR) – Table 3.

**Table 3:** QoL life scores assessed by Short Form Health Survey (SF-36)

	Y (repair)	O (repair)	Ross	M (mechAVR)
	*n* = 36	*n* = 52	*n* = 22	*n* = 29
Physical functioning	89.4 ± 13.1	70.7 ± 22.1	88.6 ± 19.3	82.5 ± 18.0
*p*-value	Y > M	0.049		
	Y > O	0.0003		
	R > O	0.001		
Role physical	78.1 ± 33.3	55.9 ± 42.7	86.9 ± 25.1	68.3 ± 39.6
*p*-value	Y > O	0.015		
	R > O	0.002		
Bodily pain	83.9 ± 22.6	71.1 ± 27.5	89.2 ± 17.3	78.2 ± 19.0
*p*-value	M > O	0.044		
	R > M	0.023		
	R > O	0.022		
General health	71.4 ± 17.9	55.9 ± 18.6	71.2 ± 22.7	53.5 ± 18.7
*p*-value	Y > O	0.003		
	Y > M	0.002		
	R > O	0.005		
	R > M	0.013		
Vitality	62.4 ± 19.7	54.9 ± 15.8	62.9 ± 16.1	54.8 ± 20.4
*p*-value	Y > O	0.015		
	R > O	0.006		
	R > M	0.046		
Social functioning	83.3 ± 22.6	74.3 ± 21.1	88.1 ± 21.3	77.4 ± 23.0
*p*-value	Y > O	0.026		
	R > O	0.004		
Role emotional	83.3 ± 31.9	65.7 ± 40.5	85.7 ± 28.3	73.1 ± 32.1
N.S.				
Mental health	72.4 ± 18.6	70.1 ± 16.9	74.3 ± 16.0	65.8 ± 17.1
N.S.				

The specific valve related questionnaire was also completed by 139 patients (Y = 36, O = 52, R = 22, M = 29). The response distribution with regard to 8 questions is shown in Table 4. In response to four questions (impact of follow-up care, visiting a physician, blood tests and awareness of risk of complications) there were no significant differences among the non-replacement responders (Y, O, R). When these three groups were pooled together and compared to the M group they showed significantly greater freedom of valve-related life-style limitations. Patients in the mechanical AVR group (M) were more concerned by the risk of bleeding due to medication than the non-replacement patients. In regard to the possible risk of reoperation, the younger repair patients (Y) were more disturbed than the older ones (O).

**Table 4:** QoL expressed in relation to specific valve related concerns, acc. Perchinsky (modif.) [12]

	Y (repair)	O (repair)	Ross	M (mechAVR)
	*n* = 36	*n* = 52	*n* = 22	*n* = 29
	(%)	(%)	(%)	(%)
1. If you had to do it over again, would you chose the same procedure?				
Yes	92	71	82	72
I don’t know	6	21	18	21
No	3	8	0	7
*p* value - N.S.				
2. After the valve surgery, are you annoyed by the need of follow-up care?				
Never/rarely	89	87	91	69
Occasionally	11	13	5	31
Frequently/always	0	0	0	0
*p* value Y vs O vs R - N.S.				
(Y + O + R) vs M - 0,016				
3. After the valve surgery, are you annoyed by visiting the doctor frequently?				
Never/rarely	86	73	77	52
Occasionally	14	27	23	48
Frequently/always	0	0	0	0
*p* value Y vs O vs R - N.S.				
(Y + O + R) vs M - 0,0088				
4. Are you annoyed by frequent blood tests?				
Never/rarely	92	77	86	34
Occasionally	6	19	9	52
Frequently/always	3	4	5	14
*p* value Y vs O vs R - N.S.				
(Y + O + R) vs M - 0,00001				
5. Are you disturbed the possibility of complications due to your implanted valve?				
Never/rarely	42	58	64	24
Occasionally	53	42	27	66
Frequently/always	6	0	9	10
*p* value Y vs O vs R - N.S.				
(Y + O + R) vs M - 0,0079				
6. Are you disturbed by the risk of bleeding due to medication?				
Never/rarely	78	73	86	31
Occasionally	8	27	9	55
Frequently/always	14	0	5	14
*p* value Y vs O vs R - 0,008				
(Y + O + R) vs M - 0,00001				
7. Are you disturbed by the risk of valve failure?				
Never/rarely	53	65	68	55
Occasionally	39	33	27	38
Frequently/always	8	2	5	7
*p* value - N.S.				
8. Are you disturbed by the risk of reoperation?				
Never/rarely	28	60	59	41
Occasionally	56	31	32	52
Frequently/always	17	10	9	7
*p* value Y vs O - 0,013				

## Discussion

Aortic valve-sparing surgery is an attractive and challenging surgical option which requires a focused interest and training of a dedicated surgeon. Active attitude in attempting the valve salvage is based on the surgeon’s belief that reconstruction of the aortic valve opens an opportunity to the patient to live without the burden of long-term anticoagulation and its related risks, which may provide a superior quality of life. This rationale should support the decision for repair against the potential risk of perioperative failure (need for re-clamping) or repair failure in postoperative period. In the recent decade, refinement of surgical techniques and systematical methodological portfolio of steps has enabled to perform aortic valve repair with a high prediction of success. As a result, there is a continuum of aortic valve repair candidates in whom three subpopulations can be identified. First, very young adult patients and women with child bearing potential form the group with strongest undebatable urge for valve conservation. The second group concerns mid-aged (45–55 years) patients who are facing the issue of sudden change of the life-style related with the onset of anticoagulation due to mechanical AVR. The third group comprises older patients whose aortic valve morphology would allow a repair, but otherwise qualify reasonably for a bioprosthesis (60–65 years of age).

Beside the known low procedural risk and good clinical outcomes [1–8, 10, 13, 14], the expectation of a superior quality of postoperative life might play a role in decision for aortic valve-sparing strategy. Hypothetically, better quality of life should result from the absence of anticoagulation and related life-style limitations, awareness of permanent risk of thromboembolic and bleeding complications, absence of blood checking and vigilance against prosthetic infection [15]. On the other hand, the fear of a potential reoperation may affect negatively the quality of life after valve-sparing aortic surgery.

Quality of life is a “soft” variable that is not easy to measure. In our study, we used a combination of two survey tools: a recognized and validated SF-36 questionnaire for a general assessment of QoL and a valve-specific questionnaire describing the specific concerns of a patient after valve surgery [11, 12].

Based on SF-36 results, from the three groups of a comparable age (Y, R and M, mean age 36, 38, 40 years, resp.) the younger valve repair patients and Ross patients scored significantly better than the patients after mechanical valve replacement in all four physical subscales and at least in two of the four mental subscales. The older patients after valve-sparing surgery (mean age 59 years) did not match the younger repair patients and had scores comparable to the patients after mechanical AVR. The most intuitive explanation for these results (physical scores in particular) is the age difference.

The results of the valve-specific questionnaire yield a different insight into the quality of life after valve surgery. Despite even an age difference, all three groups without mechanical replacement device enjoyed greater freedom from the classical negative aspects related to the valve replacement surgery. When pooled together they performed better than the mechanical AVR patients in 5 of 8 questions. The risk of a valve failure remained an occasional concern for one third of respondents irrespective of the type of valve procedure. The risk of reoperation was a substantial concern for the younger valve repair patients (and surprisingly mechanical AVR patients) but not for the older repair patients neither – paradoxically – even for the Ross patients. The reason for fear of reoperation in mechanical AVR patients in contrast to less fear in the Ross patients remains unclear; we can only speculate about a distrustful perception of a “foreign” mechanical device on one hand and a vital optimism of the Ross patients believing in the best possible scenario on the other hand. Finally, to remind the role of the self-report bias, all patients were equally satisfied with the chosen type of the surgical procedure.

What information can be derived from these results? While remaining cautious due to the small sample size and the cross-sectional nature of QoL measurements, differences in QoL do exist related to the type of valve surgery. The Ross patients do generally very well after the surgery. It is a highly selected population of young active individuals who are very satisfied after having chosen and successfully overcome a difficult surgical strategy. In the light of optimal outcome and avoidance of anticoagulation they do not worry too much about the probable need for reoperation. In younger & mid-aged patients who received valve sparing aortic procedures similar scores of general health and valve-related outcomes were found. The general quality of life in older patients after valve-sparing aortic procedures was lower but related to the valve procedure these patients enjoy favorably the avoidance of anticoagulation and are even less concerned by the risk of reoperation. The patients after mechanical aortic valve replacement display consistently lower scores of QoL linked most probably to classical anticoagulation & prosthesis aspects.

While the QoL was reported to be excellent after homograft replacement for aortic endocarditis [16] and superior after Ross operation compared to the mechanical replacement [17] the data on hypothesized beneficial impact of valve-sparing procedures on QoL are very limited. Franke et al. reported in 2010 a comparison of QoL in 67 patients after aortic composite-graft replacement vs 73 patients after David I procedure. Within a 3-years follow-up, both clinical results (incidence of serious adverse events) and QoL assessed by SF-36 were inferior in the composite-graft replacement group. The differences in QoL were significant in the age groups below 50 and between 61 and 70 years [18]. In 2012, Aicher et al. published a comprehensive study comparing QoL in patients after aortic valve repair, mechanical AVR and Ross procedure. The upper age limit in all groups was 45 years. In SF-36, they observed similar scores and trend differences between the groups in favor of the valve repair and Ross patients. In terms of general and cardiac-related anxiety the levels were increased relative to published norms in all three groups without substantial differences related to the type of procedure. Valve-related concerns of the patients displayed similar distribution of answers with the same level of post-procedural satisfaction, although limitations and fears in patients after mechanical AVR on chronic anticoagulation were more frequent [19]. These results correlate with our findings; we, moreover, studied also the older population of valve repair patients who in real-life may be candidates for valve repair as well.

### Study limitations

This is a non-randomized cross-sectional study; the study population was derived from a single institutional consecutive surgical volume during similar time interval. The subgroups were defined with interest focused on the impact of the type of the valve procedure; this, however, in the real life inevitably translates in some valvular etiology differences. While every effort was invested in repairing a purely regurgitant, pliable aortic valve, the Ross procedure and mechanical AVR were undertaken in cases of unrepairable regurgitation (retracted cusps) and also a coexistent stenosis in some cases. Despite these differences the groups were deemed comparable due to their functional status and ejection fraction. No assessments of QoL were performed pre-operatively and therefore no comparison of individual changes from pre- to post-surgery was possible. No attempts were made to adjust the scores to the time elapsed since surgery nor to stratify the patients (e.g., educational status) due to the small study size. Finally, no patients were operated from a miniinvasive approach the use of which might potentially have influenced the postoperative QoL.

## Conclusions

Quality of postoperative life is influenced by the specific type of the aortic valve surgery. Procedures that enable to avoid lifelong anticoagulation do translate into a higher quality of life and greater freedom of disturbing valve-related aspects. While the Ross procedure will probably remain to be offered to a very selected population of young active individuals, aortic valve repair should be considered in all patients with suitable morphology for its good clinical results and also a beneficial impact on the quality of life. Postoperative quality of life and importance of avoiding anticoagulation from the patient´s point of view should motivate the surgeon to achieve expertise in valve-sparing techniques.

### Ethical approval

The study was approved by Institutional Ethics Committee of the Charles University Hospital, Hradec Kralove. An informed consent was an integral part of the questionnaires sent to the patients. Regarding the availability of data and materials, public deposition of the patients’ datasets does not comply with the inner policy of the authors’ institution.

## Abbreviations

AR: aortic regurgitation
AS: aortic stenosis
AVR: aortic valve replacement
AVS: aortic valve-sparing
NYHA: New York Heart Association functional classification
QoL: quality of life
